# A Toxic Conformer of Aβ42 with a Turn at 22–23 is a Novel Therapeutic Target for Alzheimer’s Disease

**DOI:** 10.1038/s41598-017-11671-6

**Published:** 2017-09-18

**Authors:** Naotaka Izuo, Chihiro Kasahara, Kazuma Murakami, Toshiaki Kume, Masahiro Maeda, Kazuhiro Irie, Koutaro Yokote, Takahiko Shimizu

**Affiliations:** 1 Department of Advanced Aging Medicine, Graduate School of Medicine, Chiba University, 1-8-1 Inohana, Chuo-ku, Chiba 260-8670 Japan; 2 Division of Food Science and Biotechnology, Graduate School of Agriculture, Kyoto University, Kitashirakawa Oiwake-cho, Sakyo-ku, Kyoto 606-8502 Japan; 3 Department of Pharmacology, Graduate School of Pharmaceutical Sciences, Kyoto University, 46-29 Yoshidashimoadachi-cho, Sakyo-ku, Kyoto 606-8501 Japan; 4 Immuno-Biological Laboratories Co, Ltd., 1091-1 Naka, Aza-Higashida, Fujioka-shi, Gumma 375-0005 Japan; 5 Department of Clinical Cell Biology and Medicine, Graduate School of Medicine, Chiba University, 1-8-1 Inohana, Chuo-ku, Chiba 260-8670 Japan

## Abstract

Immunotherapy targeting Aβ42 is drawing attention as a possible therapeutic approach for Alzheimer’s disease (AD). Considering the significance of reported oligomerized Aβ42 species, selective targeting of the oligomer will increase the therapeutic efficacy. However, what kinds of oligomers are suitable targets for immunotherapy remains unclear. We previously identified a toxic conformer of Aβ42, which has a turn structure at 22–23 (“toxic turn”), among Aβ42 conformations. This toxic conformer of Aβ42 has been reported to show rapid oligomerization and to exhibit strong neurotoxicity and synaptotoxicity. We recently developed a monoclonal antibody against the toxic conformer (24B3), which demonstrated the increase of the toxic conformer in the cerebrospinal fluid of AD patients, indicating its accumulation in AD patients’ brains. In this study, we evaluated the therapeutic efficacy of 24B3 targeting the toxic conformer in AD model mice. The intraperitoneal administration of 24B3 for 3 months improved cognitive impairment and reduced the toxic conformer levels. Notably, this treatment did not reduce the number of senile plaques. Furthermore, the single intravenous administration of 24B3 suppressed the memory deficit in AD mice. These results suggest that the toxic conformer of Aβ42 with a turn at 22–23 represents one of the promising therapeutic targets.

## Introduction

Alzheimer’s disease (AD) is a progressive neurodegenerative disease in which cognitive impairment is one of the main symptoms. The pathological hallmarks of AD include the deposition of senile plaques and neurofibrillary tangles, each of which is mainly composed of amyloid β (Aβ)^1^ and excessively phosphorylated tau^2^. The 40-mer and the 42-mer Aβ (Aβ40 and Aβ42, respectively) are produced from their precursor protein (APP) with two-step endoproteolysis by β-secretase and γ-secretase, which includes presenilin 1 or 2 (PS1 or PS2, respectively) as an activity center^3^. The resultant Aβ assembles and forms oligomers to induce neurotoxicity and synaptotoxicity^1,4^. The importance of Aβ42 in the pathogenesis of AD has been supported by numerous studies based on genetics and biochemistry^5–7^. In a recently reported clinical trial, immunotherapy with the aim of Aβ clearance achieved certain results in AD patients; a decrease in senile plaque deposition and the suppression of cognitive impairment^8^. These outcomes confirmed that Aβ is a therapeutic target for AD^8,9^. However, some problematic adverse effects, including edema and micro-hemorrhage, were observed in the clinical trials^8^; thus, there is a need to modify this treatment approach.

Aβ, a kind of intrinsically disordered proteins, is known to mainly adopt a random coil or α-helix conformation^10^, and the transition to β-sheet leads to its aggregation and oligomerization in a test tube^11–13^, which suggests the conformational diversity of Aβ in live animals. In addition, Aβ has various physiological functions and pathological functions. Physiologically, Aβ has been reported to exhibit neuroprotective^14^ and neurotrophic^15,16^ effects, and to be involved in the fine-tuning of neurotransmission^17^, the regulation of glucose homeostasis^18,19^ and immunomodulation^20–22^. Given such multiple physiological activities, the non-specific targeting of Aβ has the potential to cause adverse effects. This is assumed to be one of the causes of the clinical failure of Aβ-targeting immunotherapy for AD^23–26^. However, among the various conformations of Aβ, the conformations that are associated with the pathogenesis of AD remains to be elucidated.

**Figure 1:**
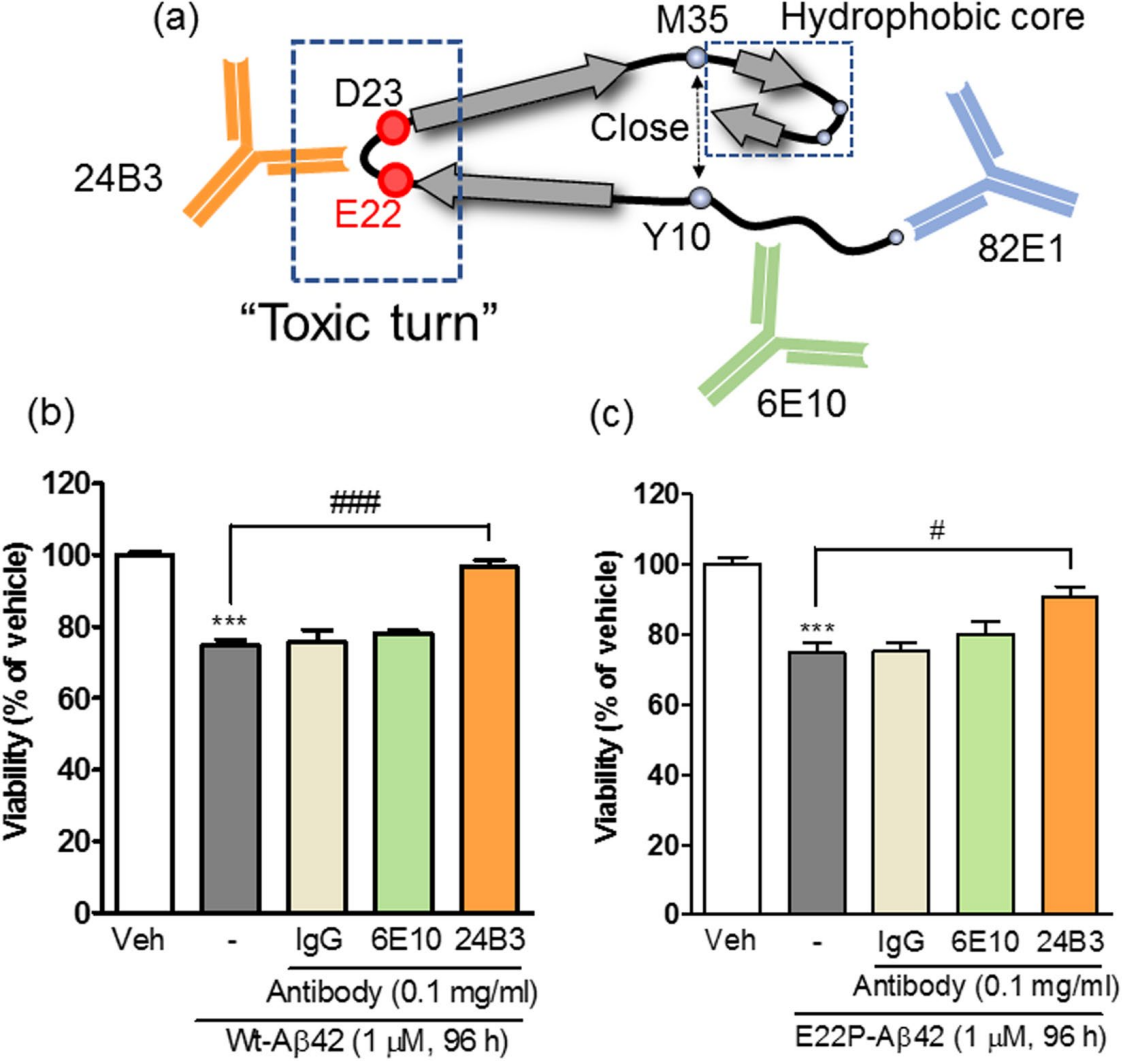
The protective effect of 24B3 on the neurotoxicity induced by Aβ42. (**a**) A schematic illustration of the structure of the toxic conformer of Aβ42, which possesses a turn structure at 22–23 (“toxic turn”). The toxic turn formation draws Y10 and M35 closer to accelerate radical transition^31^, which contributes to the formation of the hydrophobic core in the C-terminus of Aβ42^29,30^, resulting in the stable assembly as low-molecular-weight oligomers^32,43^. 24B3 was developed as a conformation-specific antibody against the toxic turn structure. The epitopes of the conventional anti-Aβ antibodies, 6E10 and 82E1, are E3-S8 and N-terminus, respectively. (**b** and **c**) The antibodies are mixed with Wt-Aβ42 (**b**) or E22P-Aβ42 (**c**) before the treatment, and the mixtures were applied to primary cortical neurons. Wt-Aβ42 and E22P-Aβ42 were applied at a concentration of 1 µM for 96 h. Antibodies were applied at a concentration of 0.1 mg/ml (approximately 0.685 µM). ***p < 0.001 vs vehicle (Veh). ^#^p < 0.05, ^###^p < 0.001.

We have been investigating the conformation of Aβ42 that is crucial for the pathogenesis of AD. Through a long series of biophysical and biochemical analyses, we identified a “toxic conformer” of Aβ42, which has a turn structure at positions 22–23 (“toxic turn”)^27–33^ and which induces quick β-sheet formation^29,32,34^, potent neurotoxicity^29,32,34–36^, and strong synaptotoxicity^35^ (Fig. 1a). Recently, three independent research groups confirmed that Aβ42 makes a turn at positions 22–23^37–39^, and Lyubchenko’s group also showed the significance of the sequence H14-D23, which mediates aggregation in the nanomolar order^40–42^. The formation of the turn at positions 22–23 brings Tyr10 and Met35 closer, thereby accelerating radical production^31^ (Fig. 1a), which contributes to the formation of the hydrophobic core in the C-terminus of Aβ42^29,30^, resulting in stable assembly as low-molecular-weight oligomers^32,43^ (Fig. 1a). For further investigation, we developed a specific antibody for the toxic conformer of Aβ42^43,44^. We obtained two representative antibodies (11A1 and 24B3) by the immunization of E22P-Aβ10–35, a minimal Aβ fragment to induce neurotoxicity, to mice and by the screening with the criteria of the positivity to the turn-forming Aβ42 mutants and the negativity to the turn-breaking mutants^43,44^ (Fig. 1a). 11A1 detected the intracellular accumulation of the toxic conformer of Aβ42 in neurons derived from the iPSCs of AD patients^45^ and in AD model mice^46^. 24B3, which shows higher specificity to the toxic conformer than 11A1^43^, captured the toxic conformer in the cerebrospinal fluid (CSF) of AD patients^43^. These observations indicate that the toxic conformer exists in the brain of AD patients. Moreover, it is noteworthy that the ratio of the toxic conformer to total Aβ42 increased in the CSF of AD patients^43^, suggesting the potential application of 24B3 in the diagnosis of AD. These results indicate the possibility that the toxic conformer of Aβ42 is a therapeutic target in AD. In this study, we investigated the effects of the administration of 24B3 to AD mice in order to evaluate this hypothesis.

## Results

### Neutralization of the toxic conformer with 24B3 suppressed the neurotoxicity induced by synthetic Aβ42

Previous studies have reported that synthetic wild-type (Wt)-Aβ42 spontaneously forms the toxic conformer, which is detected by 24B3 (Fig. 1a)^43^. We investigated the effects of 24B3 on the neurotoxicity induced by Wt-Aβ42 (Fig. 1b) and E22P-Aβ42 (Fig. 1c), which mimics the toxic conformer, on rat primary cortical cultures. E22P-Aβ42 was previously reported to draw Y10 and M35 closer and thereby induce radical transfer, leading to the stable formation of the toxic conformer of Aβ42^31^. Wt-Aβ42 and E22P-Aβ42 (1 µM, 96 h) induced significant neurotoxicity in MTT assay (Fig. 1b and c). Control-IgG showed no effects on Aβ42-induced neurotoxicity, while 24B3 almost completely suppressed the Aβ42-induced neurotoxicity (Fig. 1b and c). Similar protective effects against Aβ42 were obtained in the previous study in which 24B3 was applied to neuroblastoma SH-SY5Y cells^43^. On the other hand, another anti-Aβ antibody 6E10, the epitope of which is located at positions E3 to S8 (Fig. 1a), did not inhibit Aβ42-induced neurotoxicity; this result is supported by the report from Glabe *et al*.^47^. Moreover, 82E1, which reacts with the N-terminus of Aβ (Fig. 1a), did not show any protective effects against Aβ42-induced neurotoxicity in SH-SY5Y cultures^43^. These results suggest the significance of the toxic turn structure at 22–23 in the induction of neurotoxicity of Aβ42 and the effectiveness of targeting such a turn structure for suppressing neurotoxicity *in vitro*.

### The toxic conformer of Aβ42 is detected in the soluble fraction of the brain of AD model mice by 24B3

**Figure 2:**
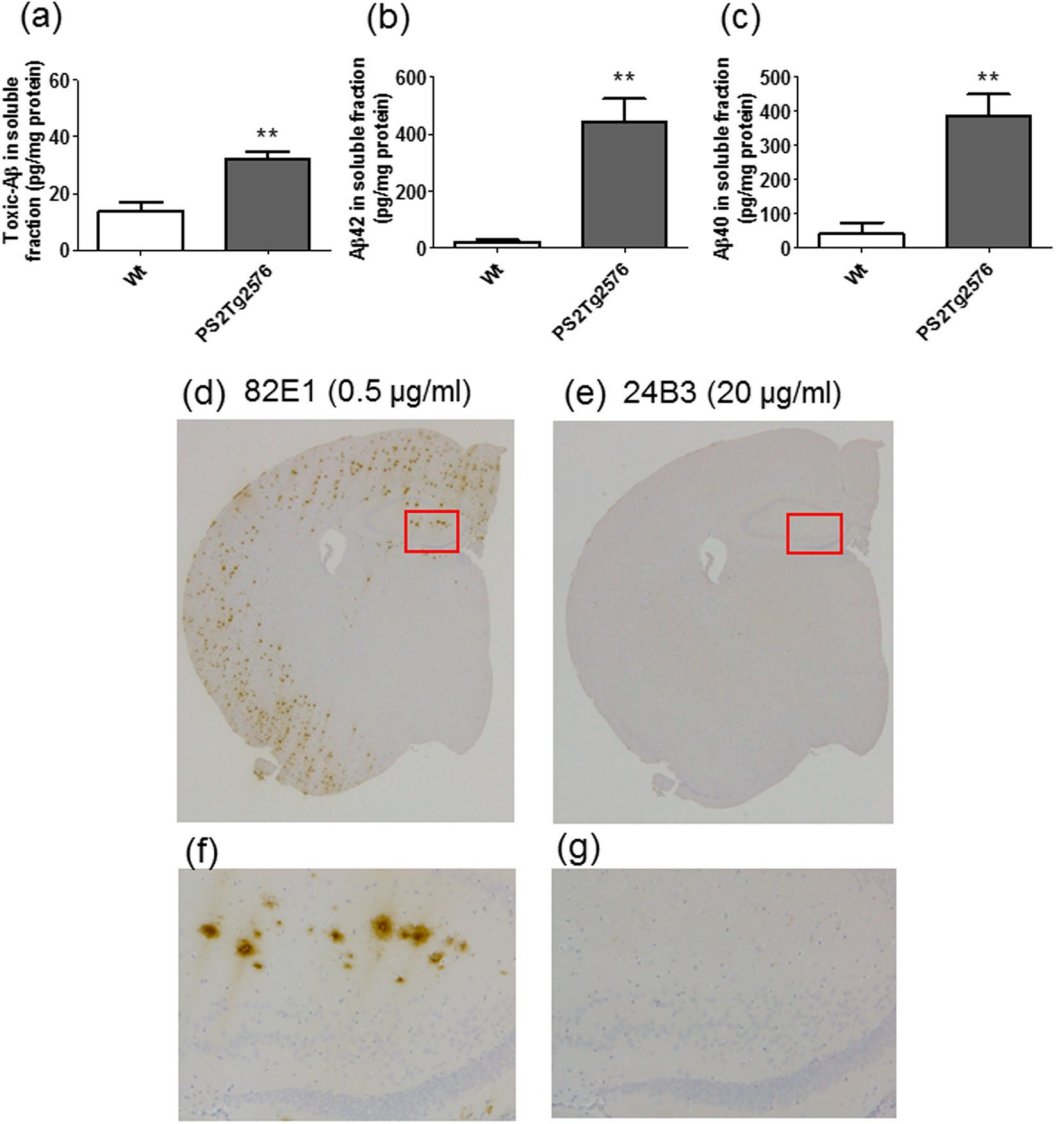
24B3 detects the toxic conformer of Aβ42 in the soluble fraction from the brain of AD model mice. (**a**–**c**) The levels of the toxic conformer of Aβ42 (**a**), total Aβ42 (**b**), and total Aβ40 (**c**) in the soluble fraction of the brain of PS2Tg2576 at 6 months of age. (**d**–**g**) Immunohistochemical staining of brain sections from PS2Tg2576 was performed with 82E1 (0.5 µg/ml) (**d** and **f**), and 24B3 (20 µg/ml) (**e** and **g**). The slices were exposed to formic acid for antigen activation. High magnification images of the area inside the red rectangles (**d** and **e**) are shown in f and g. **p < 0.01.

We recently constructed an ELISA system, with 82E1 for capturing and 24B3 for detecting, to enable us to detect and quantify the toxic conformer in the CSF from AD patients^43^. We also attempted to detect the toxic conformer in AD model mice. In this trial, we selected Tg2576, which harbors human APP with the Swedish mutation, with overexpression of human PS2 carrying the Volga German Kindred mutation (N141I)^48^, which increases the ratio of Aβ42 to Aβ40 to accumulate severely amyloid plaques in these double transgenic mice (PS2Tg2576)^49^. The toxic conformer in the brain soluble fraction prepared from PS2Tg2576 was positively detected by using our ELISA (Fig. 2a)^49^. The Aβ42 (Fig. 2b) and Aβ40 (Fig. 2c) contents in this fraction were consistent with those previously reported^49^. To investigate the presence of the toxic conformer in the senile plaques, we performed immunohistochemical staining of brain sections from AD model mice by 24B3. The numeric value of the group of Wt mice is probably a nonspecific signal in ELISA. 82E1 (0.5 µg/ml) staining clearly detected the deposition of senile plaques (Fig. 2d and f), while 24B3 (20 µg/ml) showed no staining (Fig. 2e and g). These results suggest that the toxic conformer was present in the soluble fraction, rather than in the senile plaques in AD mice. In our related study, we also found another stable conformer of Aβ42 with a turn structure at 25–26 that might be a physiological conformation and named it “non-toxic conformer”^29,32^. This turn structure at 25–26 was also observed in Aβ40 fibrils^50^. We confirmed the immunoselectivity of 24B3 between the toxic and non-toxic conformers, by immunoprecipitation (IP) without denaturing condition (Supplementary Fig. 1). In this experiment, 24B3 exhibited direct binding to monomer and oligomer of E22P-Aβ42, but not to G25P-Aβ42, a mimic of the non-toxic conformer.

### The chronic administration of 24B3 ameliorated behavioral abnormalities in PS2Tg2576

**Figure 3:**
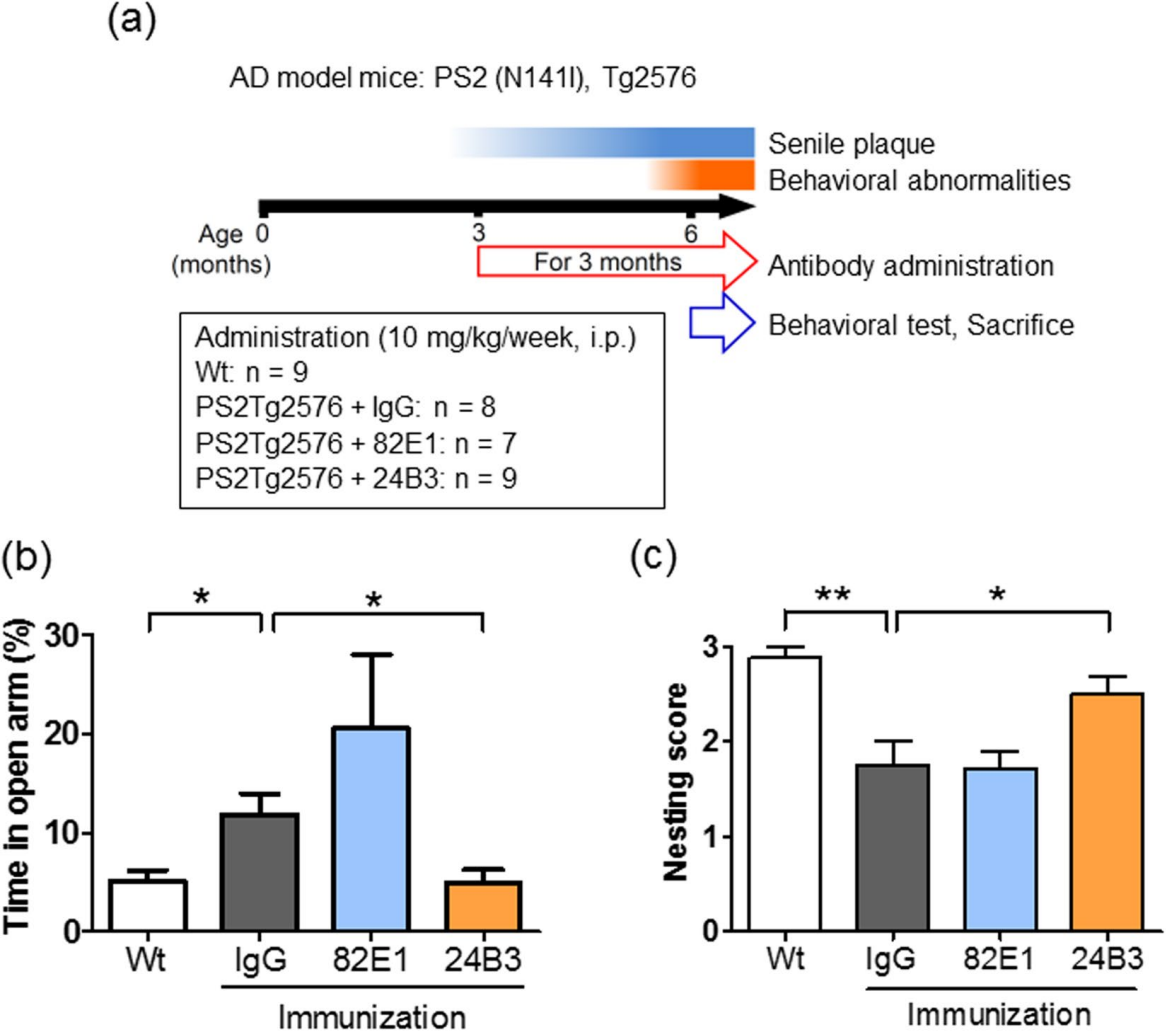
The chronic administration of 24B3 ameliorated behavioral abnormalities in PS2Tg2576. (**a**) A protocol for the chronic administration of 24B3. PS2Tg2576 exhibits senile plaque accumulation from 2–3 months of age, and cognitive impairment from 4–5 months of age. Control-IgG, 82E1 and 24B3 (10 mg/kg/week) were intraperiosteally administered to PS2Tg2576 at from 3 months to 6 months of age. At 6 months of age, the behavioral tests were performed and the mice were sacrificed. Wt mice received PBS, which was administered at the same frequency as the antibodies. The numbers of mice in each group were as follows: Wt (n = 9), PS2Tg2576 + IgG (n = 7), PS2Tg2576 + 82E1 (n = 7), and PS2Tg2576 + 24B3 (n = 8). (**b**) The suppressive effect of 24B3 on behavioral abnormalities in elevated plus maze test. 24B3-treated PS2Tg2576 spent normal time in the open arm. (**c**) The suppressive effect of 24B3 on behavioral abnormality in nest construction test. The nest building ability of 24B3-treated PS2Tg2576 was restored. **p < 0.01, *p < 0.05.

Since the presence of the toxic conformer in the brain of the AD model mice was clarified, we investigated whether the passive immunization of 24B3 could ameliorate behavioral abnormalities. PS2Tg2576 show senile plaque deposition from 2–3 months of age and cognitive impairment from 4–5 months of age (Fig. 3a)^49^. To investigate the effects of the chronic administration of 24B3, the mice underwent passive immunization from 3 months to 6 months of age and received a behavioral test at 6 months of age, and then were sacrificed. Antibodies (control-IgG, 82E1 and 24B3) were intraperitoneally administrated to PS2Tg2576 at the dose of 10 mg/kg once a week. The behavioral abnormalities of the mice were evaluated by elevated plus maze (EPM) test reflecting the spatial cognition of height (Fig. 3b) and nest construction test estimating executive function (Fig. 3c and Supplementary Fig. 2). In the EPM test, the duration that PS2Tg2576 administered control-IgG spent in the open arm was significantly longer than in Wt mice, suggesting the impairment of spatial cognition (Fig. 3b). The mice administered 24B3 stayed in the open arm for a normal length of time like Wt mice, while 82E1 did not reduce the time in the open arm (Fig. 3b). In the nest construction test, PS2Tg2576 administered control-IgG were incapable of nest building, indicating the disturbance of the executive function (Fig. 3c). Similarly to the EPM test, the administration of 24B3 restored the ability to construct a nest; the mice administered 82E1 were poor at nest building (Fig. 3c). These data suggest that the chronic administration of 24B3, but not 82E1, suppressed the impairment of spatial cognition and executive function in AD mice.

### The chronic administration of 24B3 significantly reduced the levels of the toxic conformer of Aβ42 without affecting the senile plaque pathology

**Figure 4:**
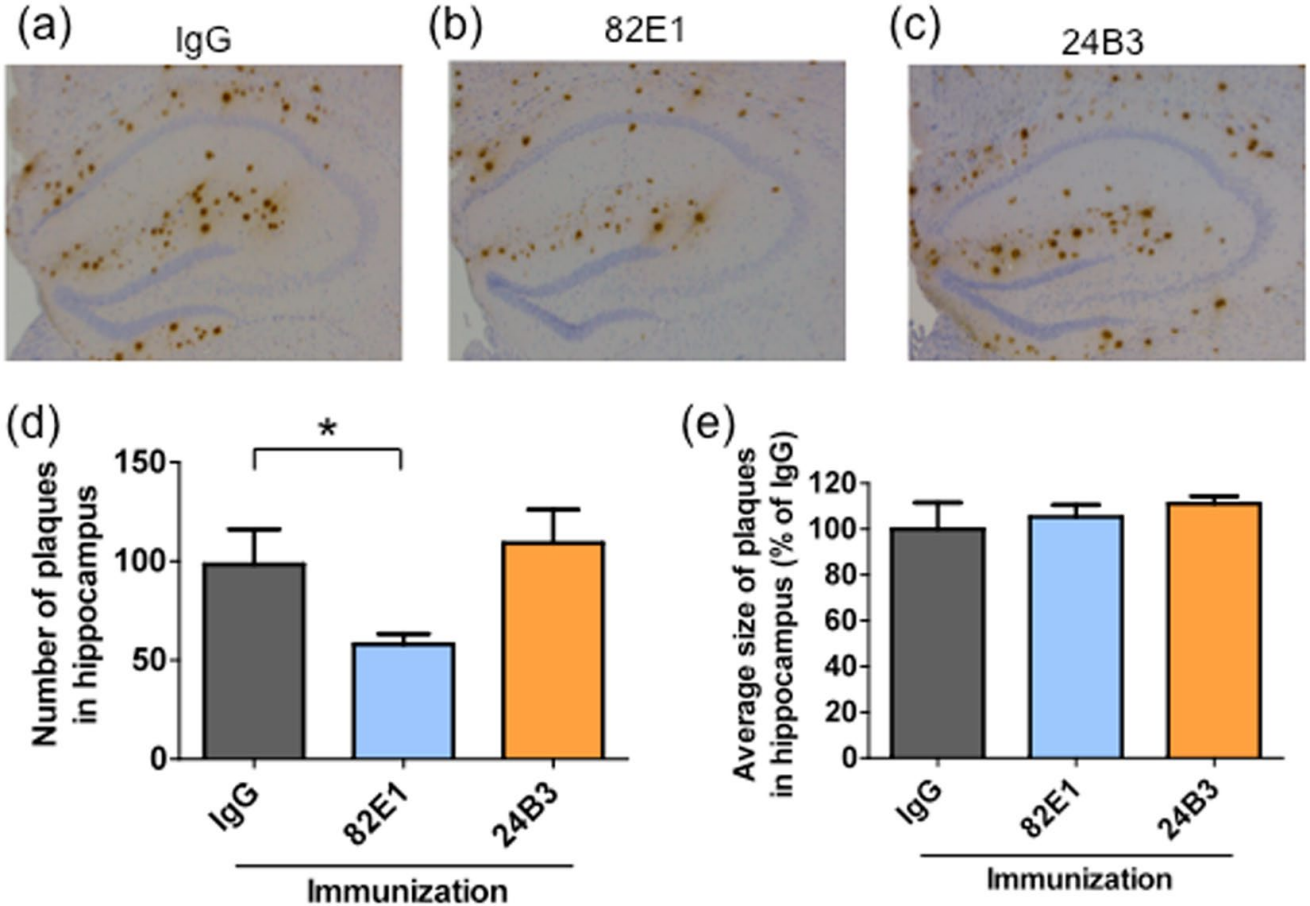
The chronic administration of 24B3 did not alter the number or size of senile plaques. Representative images of immunohistochemical staining of senile plaque by 82E1 on the hippocampus of PS2Tg2576 treated with IgG (**a**), 82E1 (**b**), and 24B3 (**c**). The average number of senile plaques in the hippocampus (**d**). The average size of the senile plaques is shown in the graph (**e**). The numbers of mice sacrificed for staining in each group were as follows: PS2Tg2576 + IgG (n = 5), PS2Tg2576 + 82E1 (n = 6), and PS2Tg2576 + 24B3 (n = 6). *p < 0.05.

**Figure 5:**
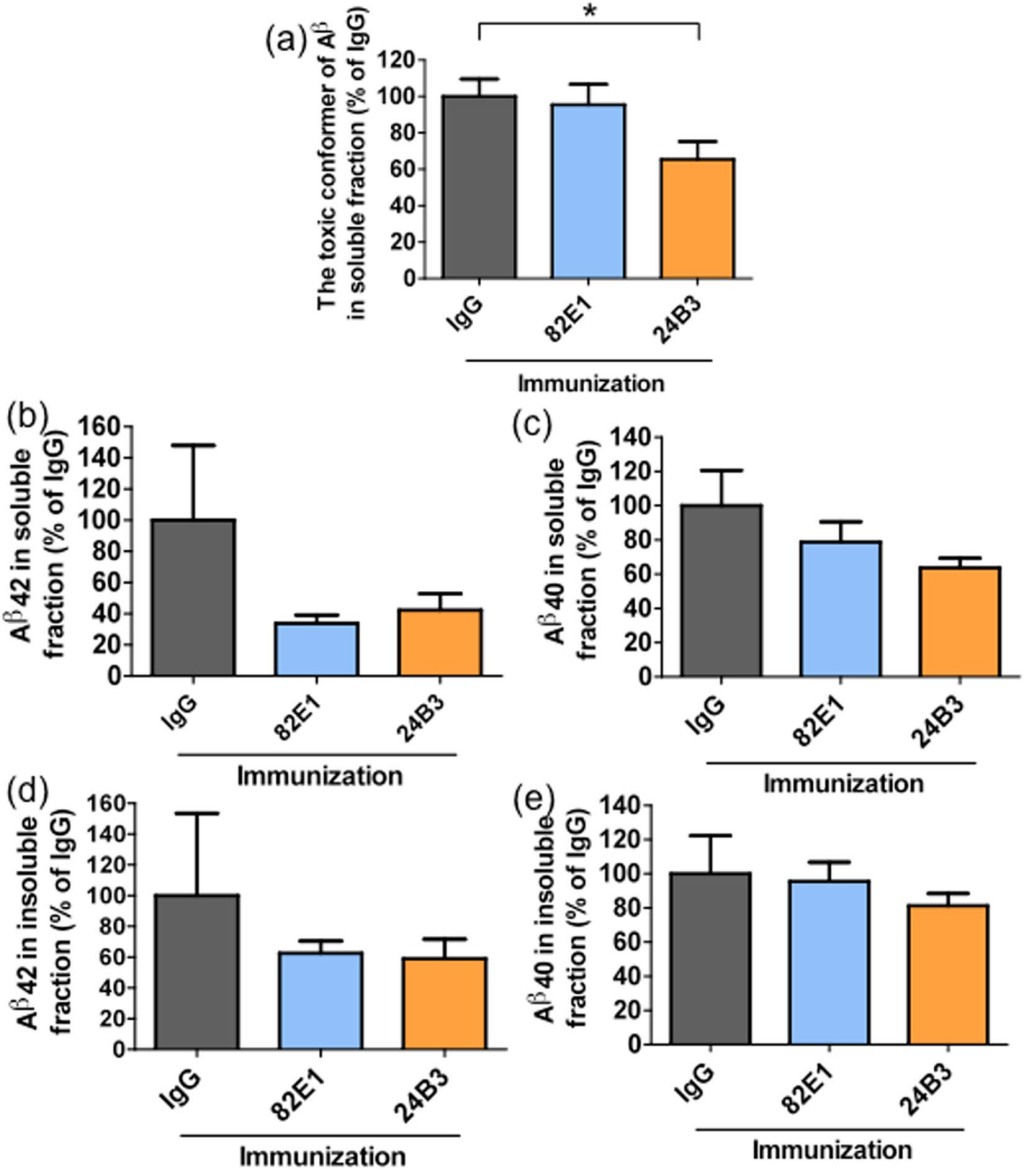
The chronic administration of 24B3 reduced the levels of the toxic conformer of Aβ42. The levels of the toxic conformer of Aβ42 (**a**), total Aβ42 (**b**), and total Aβ40 (**c**) in the soluble fraction of the brain from PS2Tg2576 treated with IgG, 82E1 and 24B3. The levels of total Aβ42 (**d**), and total Aβ40 (**e**) in the insoluble fraction of brain from PS2Tg2576 treated with IgG, 82E1 and 24B3. The numbers of mice sacrificed for measurements in each group were as follows: PS2Tg2576 + IgG (n = 5), PS2Tg2576 + 82E1 (n = 6), and PS2Tg2576 + 24B3 (n = 6). *p < 0.05.

To investigate the effects of the chronic immunization on Aβ pathology, we performed immunohistochemical and biochemical analyses. Brain sections from PS2Tg2576 with passive immunization were stained by 82E1. Passive immunization with 82E1 reduced the number of the senile plaques in PS2Tg2576 compared with the mice administered IgG (Fig. 4a,b and d). Interestingly, immunization with 24B3 did not reduce the number of plaques (Fig. 4a,c and d). There were no differences in the average size of the plaques among the three groups (Fig. 4e). Microglia play a role in clearing senile plaques by phagocytosis. Consistent with the effects of 24B3 on plaque pathology, 24B3 did not affect microglial activation (Supplementary Fig. 3). Next, we prepared soluble and insoluble brain fractions to measure the levels of Aβ. The levels of the toxic conformer in the soluble fraction were significantly reduced by the administration of 24B3, but not of 82E1 (Fig. 5a). On the other hand, there were no significant differences in the levels of the total Aβ42 (Fig. 5b and d) or Aβ40 (Fig. 5c and e) in the soluble or insoluble fractions among the IgG-, 82E1- and 24B3-treated groups. Taken together, the chronic administration of 24B3 ameliorated the cognitive deficits in PS2Tg2576 with the reduction in the levels of the toxic conformer of Aβ42, without affecting plaque pathology. In contrast, while 82E1 reduced the number of the senile plaques, it did not suppress the abnormal behaviors.

### The single administration of 24B3 ameliorated the memory impairment of Tg2576

**Figure 6:**
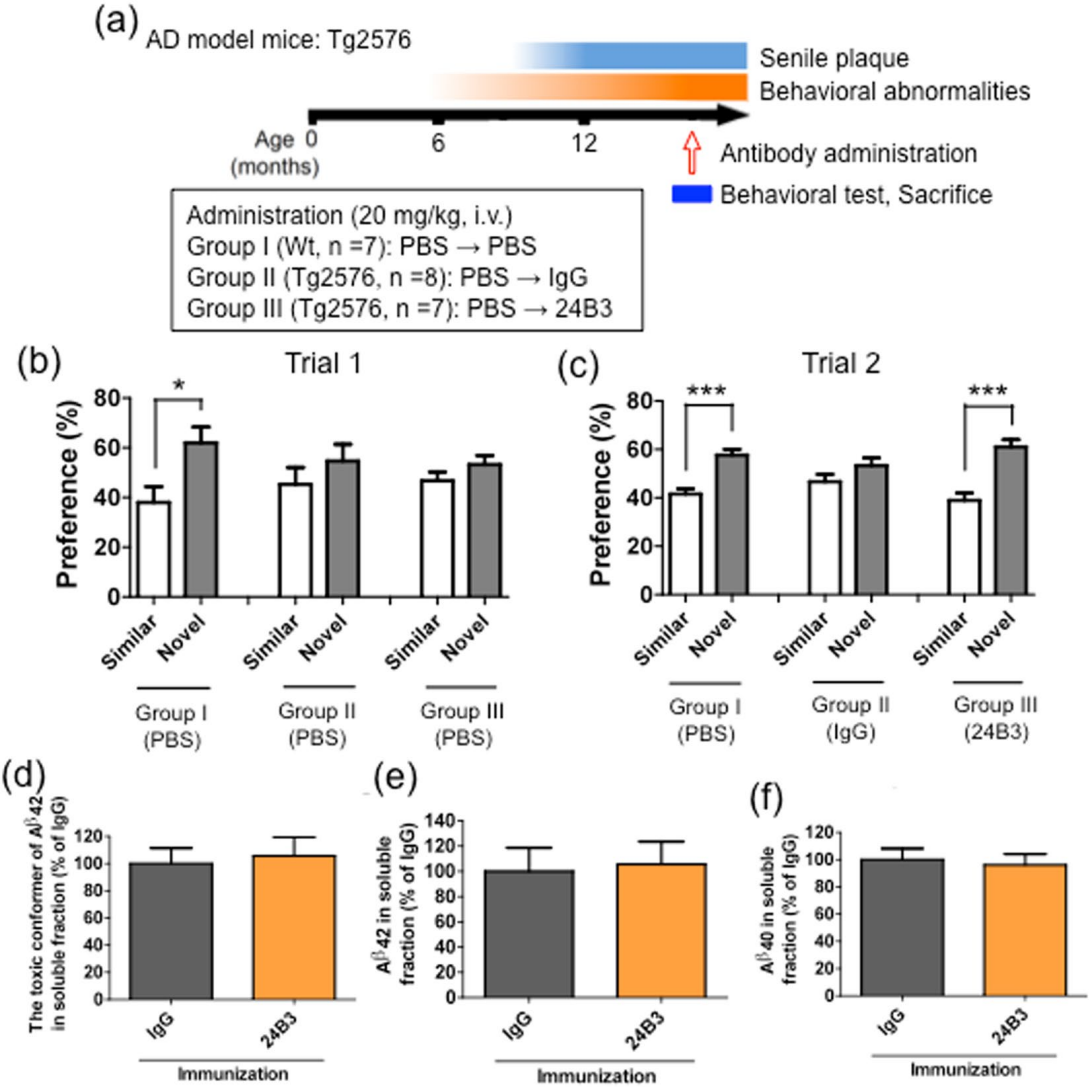
The single intravenous administration of 24B3 ameliorated memory impairment in Tg2576. (**a**) The protocol of the acute administration of 24B3. Female Tg2576 mice (16–18 months of age) received a single intravenous injection of control-IgG or 24B3 (20 mg/kg). The protocol of the novel object recognition test is detailed in Supplementary Fig. 4. (**b**) In trial 1, Wt mice and Tg2576 were injected with PBS. Wt mice exhibited a preference for the novel object; Tg2576 showed no preference. (**c**) In trial 2, Tg2576 treated with IgG exhibited no preference, while Tg2576 treated with 24B3 preferred the novel object. (**d**–**f**) The levels of the toxic conformer of Aβ42 (**d**), total Aβ42 (**e**), and total Aβ40 (**f**) in the soluble brain fraction of Tg2576 treated with IgG and 24B3. ***p < 0.001, *p < 0.05.

The experiment with the chronic regimen exhibited a suppressive effect on cognitive impairment in the AD model mice in two kinds of behavioral tests, accompanied by a reduction in the amount of toxic conformer in the soluble fraction but not in the senile plaques. These results suggest that targeting the toxic conformer is sufficient to suppress the cognitive deficit in AD model mice. Thus, we next tried another regimen to investigate the effects of the acute administration of 24B3 in aged Tg2576, another AD model mice (Fig. 6a). The memory impairment was evaluated in novel object recognition (NOR) test (Supplementary Fig. 4). In trial 1, Wt and Tg2576 mice were treated with PBS. Wt mice exhibited a preference for a novel object, while Tg2576 did not (Fig. 6b), confirming that Tg2576 exhibited memory decline. In trial 2, Wt mice were treated with PBS, while Tg2576 mice were treated with IgG or 24B3. Tg2576 that were treated with IgG exhibited no preference, while Tg2576 treated with 24B3 showed a preference for a novel object (Fig. 6c), suggesting that the single administration of 24B3 improved the memory impairment in AD mice. Surprisingly, a single dose of 24B3 did not reduce the levels of the toxic conformer (Fig. 6d), as well as those of Aβ42 (Fig. 6e) or Aβ40 (Fig. 6f) in the soluble fraction. These results suggest that only capture of the toxic turn of Aβ42 is sufficient to improve the memory function. This result is consistent with the results of the *in vitro* neutralization experiment (Fig. 1).

## Discussion

**Figure 7:**
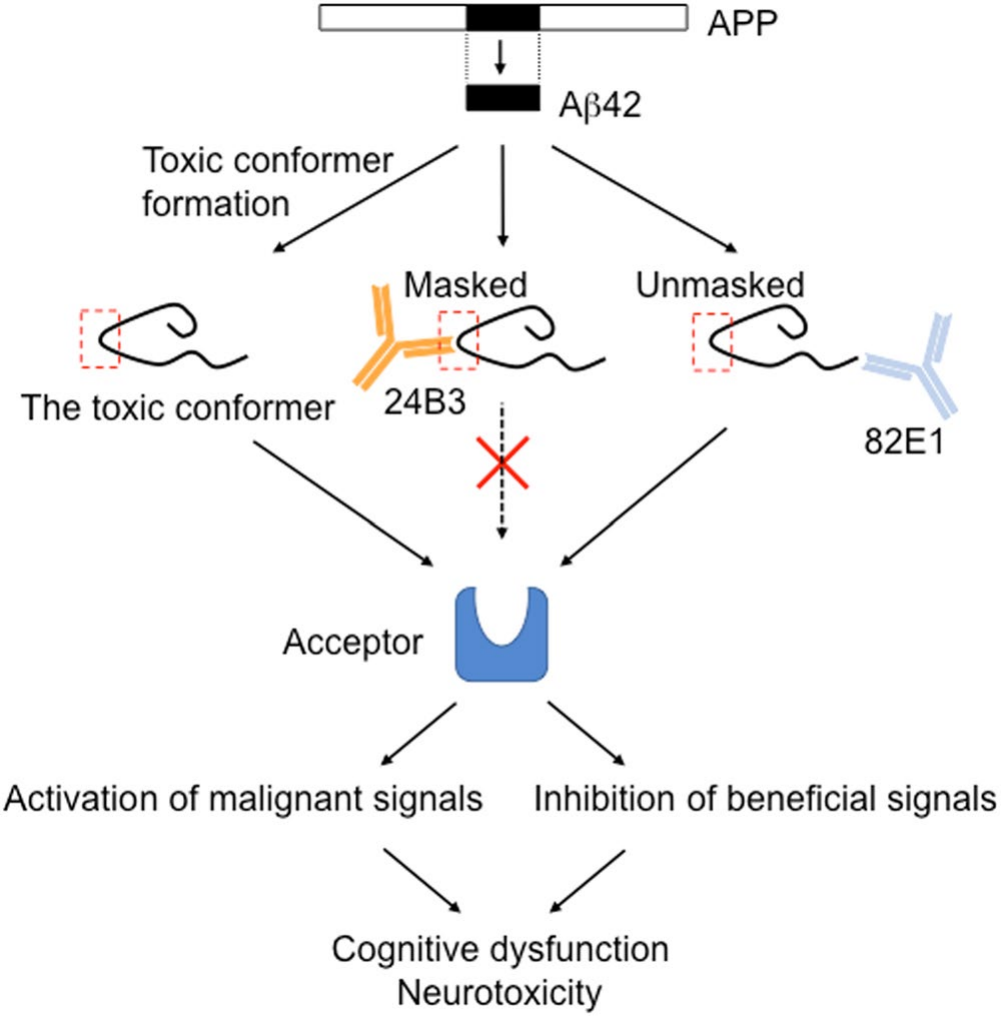
A schematic illustration of the possible protective mechanism of 24B3. The toxic conformer of Aβ42 is assumed to directly bind to a certain acceptor via the toxic turn to trigger malignant signaling. 24B3 is assumed to mask the toxic turn to block binding to its acceptor, while 82E1 does not block binding. Thus, 24B3, but not 82E1, protects against cognitive impairment and neurotoxicity.

The toxic conformer of Aβ42 in PS2Tg2576 was not detected in senile plaques and was detected in the soluble fraction by ELISA with 24B3 (Fig. 2), indicating that the toxic conformer mainly exists in the soluble fraction in AD brains. These findings do not contradict with the clinical evidence showing that the toxic conformer is detected in the CSF from AD patients^43^. An Aβ42 mutant with a deletion at position 22, Osaka mutant, which is discovered as a familial mutation that is associated with Alzheimer’s-type dementia^51^, is suggested to preferably form the toxic conformer and soluble oligomer^35^; the patients and transgenic mice with this mutation do not accumulate senile plaques^51,52^. This evidence also supports our hypothesis that the toxic conformer mainly exists in a soluble oligomer. The chronic administration of 24B3 improved the executive dysfunction and spatial cognitive impairment of AD model mice (Fig. 3), and significantly reduced (by ~40%) the levels of the toxic conformer in the soluble fraction (Fig. 5) without affecting the plaque pathology (Fig. 4). In contrast, the administration of 82E1 (the anti-N terminus of Aβ42) by the same protocol did not suppress abnormal behaviors, even though it reduced the number of plaques (Figs 3 and 4). These results did not contradict the results showing that the localization of the toxic conformer was limited to the soluble fraction, rather than the plaques (Fig. 2). Aβ oligomers are classified into two groups: those oriented to senile plaques (“on-pathway”), and those not oriented (“off-pathway”); the toxic oligomer derived from the toxic conformer might be an off-pathway oligomer^53^. These findings raise the important suggestion that the toxic conformer of Aβ42 in the soluble oligomer fraction, not in senile plaques, is a promising target to improve the cognitive impairment associated with AD, and that the therapeutic target should be the soluble oligomers with toxic conformation, not the plaques (Fig. 7).

So far, a series of anti-Aβ antibodies have been evaluated in the clinical trials and most of them failed to show their effectiveness because of the lack of potency or unignorable side effects^23–26^. The late intervention and inaccuracy of the clinical diagnosis have been suggested as reasons for clinical failure. In addition to these points, it is necessary to re-evaluate the epitope of each antibody to ensure the efficacy of therapies. Despite the apparent reduction of the number of senile plaques, the administration of bapineuzumab, the epitope of which is the Aβ sequence of D1-R5, did not result in a clinically significant cognitive improvement^23,26,54,55^. These outcomes are similar to our results from 82E1 administration (Figs 3 and 4). This may be because most of the antibodies are occupied by the Aβ monomers rich in AD brains, and the remaining antibodies are unable to capture a sufficient volume of toxic Aβ oligomers, including the toxic conformer. In the EPM test, 82E1 resulted in a non-significant worsening trend in cognitive impairment. This may be derived from the removal of the monomeric Aβ42, which is reported to modulate the neuronal signal transmission. A number of antibodies against the N-terminus of Aβ42 are reported to suppress the behavioral abnormalities in AD model mice^56^. In this study, 82E1 reduced the number of senile plaques in PS2Tg2576 by only about half. Since this effect is not as strong as in other previously reported antibodies^56^, the failure of 82E1 to suppress the behavioral abnormalities does not contradict the findings of previous studies^56^. Furthermore, solanezumab, which is a “sequence-specific” antibody to H13-K28, did not improve cognitive impairment in clinical trials^24,25^. It was reported that solanezumab is likely to capture monomeric Aβ, thus this antibody probably has low affinity to the toxic turn of the toxic conformer. This may be one reason for the clinical failure. In our previous observations, 4G8, which binds to a specific “sequence” of L17-V24, did not show any protective effect against Aβ42-induced neurotoxicity in primary cortical neurons^57^ or a neuroblastoma cell line^43^. This is probably because 4G8 exhibits low-binding capacity to the turn structure of the toxic conformer^36^. One of the determinants of the clinical potency of AD therapies may be the specificity and reactivity to the toxic turn of Aβ42, not the affinity to senile plaques. This notion is supported by the fact that there was little correlation between the clinical severity of AD and the Aβ plaque burden in human patients^58,59^ and by the fact that there are cases in which patients have no clinical abnormalities despite plaque accumulation in the brain^60–62^.

In the chronic protocol, targeting the toxic conformer in the soluble fraction relieved cognitive dysfunction without affecting the number of senile plaques (Figs 3 and 4). Given that half-life of Aβ is almost half an hour^63,64^, there was a possibility that the acute administration of 24B3 is effective for cognitive recovery. In fact, in our investigation, only the single intravenous administration of 24B3 ameliorated the memory deficit in NOR test (Fig. 6). Surprisingly, this amelioration of memory impairment was not accompanied by a reduction in the levels of the toxic conformer (Fig. 6). In the *in vitro* experiment, 24B3 inhibited Aβ42-induced neurotoxicity (Fig. 1b and c). This probably occurred without a decrease in the levels of the toxic conformer in the medium, which is consistent with the results observed after the acute administration *in vivo*. These data imply that the expression of the acute beneficial effects of 24B3 does not necessarily require the reduction of the toxic conformer itself. Although the mechanism underlying the malignant effect of the toxic conformer remains to be elucidated, it is easy to assume that the toxic conformer binds to certain acceptor proteins, which convey the harmful signaling to induce synaptotoxicity and neurotoxicity, or inhibits the beneficial signaling for the cell survival or neural transmission (Fig. 7). Given that the direct binding of 24B3 to the toxic conformer was confirmed by the IP methods (Supplementary Fig. 1), as reported previously^43^, the protective effect of 24B3 is assumed to be mediated by the prevention of binding between the toxic conformer and its acceptors, rather than by the clearance by immune cells (Fig. 7). The clinical trials of anti-Aβ immunotherapy revealed that clearance of the Aβ burden on microvasculature can induce edema or micro-hemorrhage, so-called amyloid-related imaging abnormalities (ARIA)^65^, which is a common adverse effect of these types of immunotherapy. Even though aducanumab succeeded in deaccelerating the cognitive decline in mild to moderate AD patients, this antibody could not avoid these kinds of side effects derived from the potent capacity to clear the Aβ burden^8^. Aducanumab was reported to show enhanced microglial recruitment toward senile plaques^8^, while 24B3 did not show any additional activation of microglia (Supplementary Fig. 3). From this point of view, 24B3 may be associated with fewer adverse effects including ARIA.

Memory process is composed of multistage, such as acquisition, consolidation, retention, and retrieval; and memory is stored in the engram cells in hippocampus^66^. A recent study reported that AD mice exhibited impairment in their retrieval of stored memory information before the plaque accumulation^67^, and that the activation of the memory engram cells improved this impairment^67^. In NOR test in the present study, each antibody was administered for three consecutive days after the acquisition phase. It is suggested (based on this experimental procedure) that 24B3 affects the retrieval stage of the memory process and that the toxic conformer is involved in the impairment of memory retrieval in AD model mice. Furthermore, we previously reported that the toxic conformer strongly inhibits long-term potentiation (LTP)^35^, which is a key step in memory acquisition^68,69^, and which is disturbed in AD^70^. Taken together with this evidence, it is suggested that the toxic conformer of Aβ42 plays several crucial roles in the memory impairment.

The present study put the toxic conformer forward for the promising therapeutic strategy for AD. In AD mice, 24B3 showed sufficient specificity and affinity to the toxic conformer to ameliorate the cognitive impairment. In the case of AD patients, however, the therapeutic antibodies might require higher specificity and affinity. The clinical potency of 24B3 should be evaluated, and efforts are expected to be made to develop antibodies with high selectivity and affinity to the toxic conformer. In addition to immunotherapy, there are other approaches that can target the toxic conformer. The antagonism of the acceptors of the toxic conformer and the structural destabilization of the conformer are also appealing strategies. The elucidation of the mechanism underlying the development of cognitive impairment and neurotoxicity mediated by the toxic conformer is the next task to facilitate further drug development. The neutralization of the toxic conformer could lead to effective AD therapies with fewer adverse effects.

## Methods

### AD model mice

Tg2576 were purchased from Taconic Laboratories. PS2Tg2576 were developed by crossbreeding with Tg2576 and human mutant PS2 (N141I) transgenic mice, which is previously generated as previously described^49^. Tg2576, PS2Tg2576 and littermate Wt mice were maintained in a 24 ± 1 °C room, with 55% ± 10% relative humidity, under a 12 h light/dark cycle, with *ad libitum* access to food. All experimental procedures were performed in accordance with specified guidelines for the care and use of laboratory animals, and were approved by the Animal Care and Use Committee of Chiba University.

### Antibody administration

24B3, the monoclonal antibody against the toxic conformer, was obtained as previously described^43,44^. In brief, 24B3 was obtained by the immunization of mice with G9C, E22P-Aβ9–35, followed by repeated selection with the criteria of positivity to the turn-forming mutant Aβ42 and negativity to the turn-breaking mutant Aβ42^43,44^. 82E1 was developed as an antibody against the N-terminus of Aβ. As a control-IgG, we chose mouse monoclonal antibody against keyhole limpet hemocyanin (Immuno-Biological Laboratories). Both isotypes of the antibodies are IgG1.

Each antibody was diluted with PBS to adjust the concentration. The injection volume was 10 ml/kg of body weight. In the chronic protocol, each antibody was administered weekly to male and female PS2Tg2576 by intraperitoneal injection starting from 3 months of age for as long as 3 months at a dose of 10 mg/kg. In the acute protocol, each antibody was singly injected into the tail vein of female Tg2576 at 16–18 months of age at a dose of 20 mg/kg. Wt mice recieved injection of PBS with the same frequency as antibodies.

### Elevated plus maze (EPM) test

Spatial cognition was evaluated by the EPM test, as described previously^71,72^. The maze apparatus (Muromachi Kikai) consists of four arms (30 × 6 cm) and a central square (6 × 6 cm), all of which are located as high as 40 cm above the floor. Two arms are closed ones surrounded by vertical walls of 14 cm in height; the other two are open arms without walls. In the test, mice were placed alone on the central square and were allowed to explore freely for 10 min. The motions of the mice were automatically recorded with DV-Track Video Tracking System (Muromachi Kikai).

### Nest construction test

The executive function was evaluated in nest construction test, the protocol of which was modified from a previous report^73^. Paper towel (Product number 37115, Nippon Paper) was used as the nest material. Ten pieces of paper (6 cm × 6 cm) were placed in the center of a clean cage (16.8 cm × 29.9 cm × 13.3 cm) and then mice were singly put in the cage. The nest building was assessed 4 days later according to the following criteria (Supplementary Fig. 2): score 0, no nesting or paper was scattered; 1, all paper was collected in a corner; score 2, all paper was collected in a corner and bitten or torn; 3, complete nesting was observed.

### Novel object recognition (NOR) test

Long-term memory was evaluated by NOR test (Supplementary Fig. 4). Three experimental groups (Group I-III) were made from Wt and Tg2576. Before the test, the mice were placed into an open box (45 cm × 45 cm × 15 cm) for 10 min for 5 consecutive days to allow habituation. In this study, NOR test consisted of 2 trials. In trial 1, the mice were placed facing two similar objects (P and P’) for 10 min for 3 successive days (acquisition phase); the next day, Group I-III received an intravenous injection of PBS. After 24 h, mice were placed facing object P and a novel object (object Q) for 10 min (test phase). In trial 2, the mice were placed facing two similar objects (R and R′) for 10 min for 3 successive days, and next day, Group I received an injection of PBS, while Groups II and III received IgG or 24B3. After 24 h, the mice were placed facing object R and a novel object (object S) for 10 min, and then sacrificed. In the test phase, the number of times the mice touched the objects with their nose was counted. The preference for the novel object was scored as the ratio of touches of the novel object to the total number of touches. It was confirmed that the mice had no significant preference for any of the objects. Any mice that showed a total touch count of <5 were excluded from the experiment.

### Brain sampling

The mice were sacrificed at 48 h after the final administration of the antibody in the chronic protocol, and immediately after the behavioral experiment in the acute protocol. Under pentobarbital anesthesia (intraperitoneal injection), the mice were perfused with PBS to remove their blood and then their brains were collected. One hemisphere was frozen with liquid nitrogen for the biochemical assays; the other was soaked in 4% paraformaldehyde (Nacalai tesque) for fixation for immunohistochemical staining.

### Aβ ELISA

Frozen brain hemispheres were homogenized in TBS with protease inhibitors (Complete mini, Roche), 0.7 µg/ml pepstatin A, and 1 mM phenylmethylsulphonyl fluoride. After the centrifugation (55,000 × g, 30 min, 4 °C) of the homogenate, the resultant supernatant was collected as a soluble fraction. The pellet was suspended in 6 M guanidine-HCl (Wako) and centrifuged (55,000 × g, 30 min, 4 °C) to collect the supernatant as an insoluble fraction.

The concentrations of the toxic conformer of Aβ42, total Aβ42, total Aβ40 in the soluble and insoluble fractions were measured by ELISA (Cat# 27709, 27719, and 27718, respectively, Immuno-Biological Laboratories) in accordance with the manufacturer’s instructions.

### Immunohistochemistry

Five micrometer-thick coronal paraffin-embedded sections were prepared from fixed brain hemispheres. After deparaffinization and hydration, the slices were treated with formic acid (Nacalai Tesque) for 30 s for antigen activation. To inhibit the endogenous peroxidase, the brain sections were soaked in methanol with 0.1% H_2_O_2_ for 30 min. After washing with ice-cold PBS containing 0.02% Tween-20 (PBST), blocking was performed in blocking buffer, PBST with 10% goat serum (Sigma), for 30 min at room temperature. The first antibody, 82E1 (0.5 µg/ml) or 24B3 (20 µg/ml), diluted by blocking buffer was applied overnight at 4 °C. After washing with PBST, the second antibody, biotinylated mouse IgG (Vector Laboratories), diluted by blocking buffer was applied for 1 h at room temperature. The immunological signals were enhanced using an avidin biotin complex reaction kit (Vector Laboratories) including HRP-linked avidin. To visualize the signals, brain sections were treated with 3,3′-diaminobenzidine (Dojindo) solution in TBS with 0.1% H_2_O_2_. For reference, nuclei were stained with 4′,6-diamino-2-phenylindole (Dojindo). After dehydration and soaking in xylene, the brain sections were mounted with a coverslip and a reagent (Millipore).

### MTT assay on rat primary neurons

The experimental procedures were approved by the Kyoto University Animal Experimentation Committee. Neuronal cultures were prepared from the cerebral cortices of fetal Wistar rats (Nihon SLC) at 17–19 days of gestation. The cultures were maintained in Neurobasal medium (Life Technologies) with 2% B-27 supplement (Life Technologies), 25 mM sodium glutamate, and 500 µM L-glutamine at 37 °C in a humidified atmosphere of 5% CO_2_. On the 5th day *in vitro* (DIV), the medium was replaced with sodium glutamate-free Neurobasal medium. Mature cultures (DIV8–12) were used for the experiments. In all experiments, B-27 supplement without antioxidants was used during the Aβ42 treatment.

Neurotoxicity was evaluated by MTT assay. Before treatment, Aβ42 was dissolved in PBS with 0.1% NH_4_OH, incubated for 30 min on ice, and then the Aβ42 solution was diluted 10 times with Neurobasal medium to adjust to the final concentration of 1 µM. After 96 h of treatment, the culture medium was replaced with medium containing 0.5 mg/ml MTT (Nacalai Tesque), and the cells were incubated for 30 min at 37 °C, followed by replacement with 2-propanol to lyse the cells. The absorbance at 595 nm was measured with a spectrometer (Microplate reader model 680, Bio-rad). The medium for vehicle treatment contained 0.01% NH_4_OH.

### Statistical analyses

All data are presented as the mean ± s.e.m. The differences were analyzed with one-way ANOVA followed by Tukey’s test or unpaired Student’s *t*-test. *P* values < 0.05 were considered to indicate statistical significance.

### Data Availability Statement

The datasets generated during the current study are available from the corresponding author on reasonable request.

## Electronic supplementary material

supplementary figures
