# Supplementary material for: A Toxic Conformer of Aβ42 with a Turn at 22–23 is a Novel Therapeutic Target for Alzheimer’s Disease
Source: Sci Rep. 2017 Sep 18;7:11811. doi: 10.1038/s41598-017-11671-6 (PMC5603611; doi:10.1038/s41598-017-11671-6)
Supplement: Supplementary file 1 — supplementary figures [file 41598_2017_11671_MOESM1_ESM.pdf]

Supplementary Information

A Toxic Conformer of Aβ42 with a Turn at 22-23 is a Novel Therapeutic Target for Alzheimer's Disease

Naotaka Izuo[1], Chihiro Kasahara[1], Kazuma Murakami[2], Toshiaki Kume[3], Masahiro Maeda[4], Kazuhiro Irie[2], Koutaro Yokote[5], Takahiko Shimizu[1†]

[1]*Department of Advanced Aging Medicine, and* [5]*Department of Clinical Cell Biology and Medicine, Graduate School of Medicine, Chiba University, 1-8-1 Inohana, Chuo-ku, Chiba, 260-8670, Japan*

[2]*Division of Food Science and Biotechnology,, Graduate School of Agriculture, Kyoto University, Kitasirakawa Oiwake-cho, Sakyo-ku, Kyoto, 606-8502, Japan*

[3]*Department of Pharmacology, Graduate School of Pharmaceutical Science, Kyoto University, 46-29 Yoshidashimoadachi-cho, Sakyo-ku, Kyoto, 606-8501, Japan*

[4]*Immuno-Biological Laboratories Co, Ltd., 1091-1 Naka, Aza-Higashida, Fujioka-shi, Gumma, 375-0005, Japan*

[†] Corresponding author

Takahiko Shimizu, PhD., e-mail: shimizut@chiba-u.jp

[Contents]

Supplementary methods

Supplementary Figure 1-3

[Supplementary methods]

Immunoprecipitation

E22P-Aβ42 and G25P-Aβ42 (synthesized as previously described), were dissolved in TBS with 0.1% NH_4OH at a concentration of 200 μM on ice. For the preparation of the input, E22P-Aβ42 solution (immediately after dissolution), and G25P-Aβ42 solution (after incubation at 37°C for 24 h), were diluted 10 times in TBS (final concentration 20 μM). A 50 μl aliquot of Streptavidin Mag Sepharose Beads (GE Healthcare), the buffer of which was replaced with TBS, was gently mixed with 0.4 μg/ml of each biotinylated antibody (control-IgG, 82E1, and 24B3) in 300 μl of TBS for 60 min at room temperature. After washing with TBS, the beads were incubated in 300 μl of input solution with gentle rotation for 2 h at 4°C. After thorough washing with PBS, the beads were incubated in 50 mM glycine (Sigma) buffer (pH 2.9) with 2 M urea (Sigma) for 5 min at 4°C to elute the immunoprecipitated Aβ. The supernatant and the input were collected and mixed with LDS sample buffer (Life Technologies) without boiling.

These samples were separated in 15% gel with tricine-SDS-PAGE in running buffer (anode buffer containing 200 mM Tris-HCl, pH8.9, and cathode buffer containing 100 mM Tricine, 0.1% SDS, 60 mM Tris, pH 8.25). The separated proteins were transferred to PVDF membrane in the blotting buffer (240 mM glycine, 30 mM Tris, 20% ethanol) in a semi-dry blotting system (Bio-rad), followed by blocking in blocking buffer, 3% non-fat skim milk and 1% bovine serum albumin in TBST. The membrane was incubated in blocking buffer containing the first antibody, 82E1 (1 μg/ml), overnight at 4°C. After washing with TBST, the membrane was incubated for 1h at room temperature in blocking buffer containing the second antibody, HRP-linked mouse IgG (Cell Signaling), diluted 2000 times. After washing, immunological signals were developed by ECL system (Bio-rad) and the images were obtained by LAS-4000 system (GE-Healthcare).

Microglial staining

Five micrometer-thick coronal paraffin-embedded sections were prepared from fixed brain hemispheres. After deparaffinization and hydration, the slices were received autoclave (121°C, 20 min) in citrate buffer (pH 4.0) for immunoactivation. After washing with ice-cold PBST, blocking was performed in blocking buffer, PBST with 10% goat serum (Sigma), for 30 min at room temperature. The first antibody, Iba-1 (5 μg/ml) diluted by blocking buffer was applied overnight at 4°C. After washing with PBS containing 0.02% Tween-20 (PBST), the second antibody, anti-rabbit IgG conjugated with AlexaFluor594 (abcam), diluted by blocking buffer was applied for 1 h at room temperature. For reference, nuclei were stained with DAPI (Dojindo). After dehydration, the brain sections were mounted with a coverslip and a reagent (Millipore).

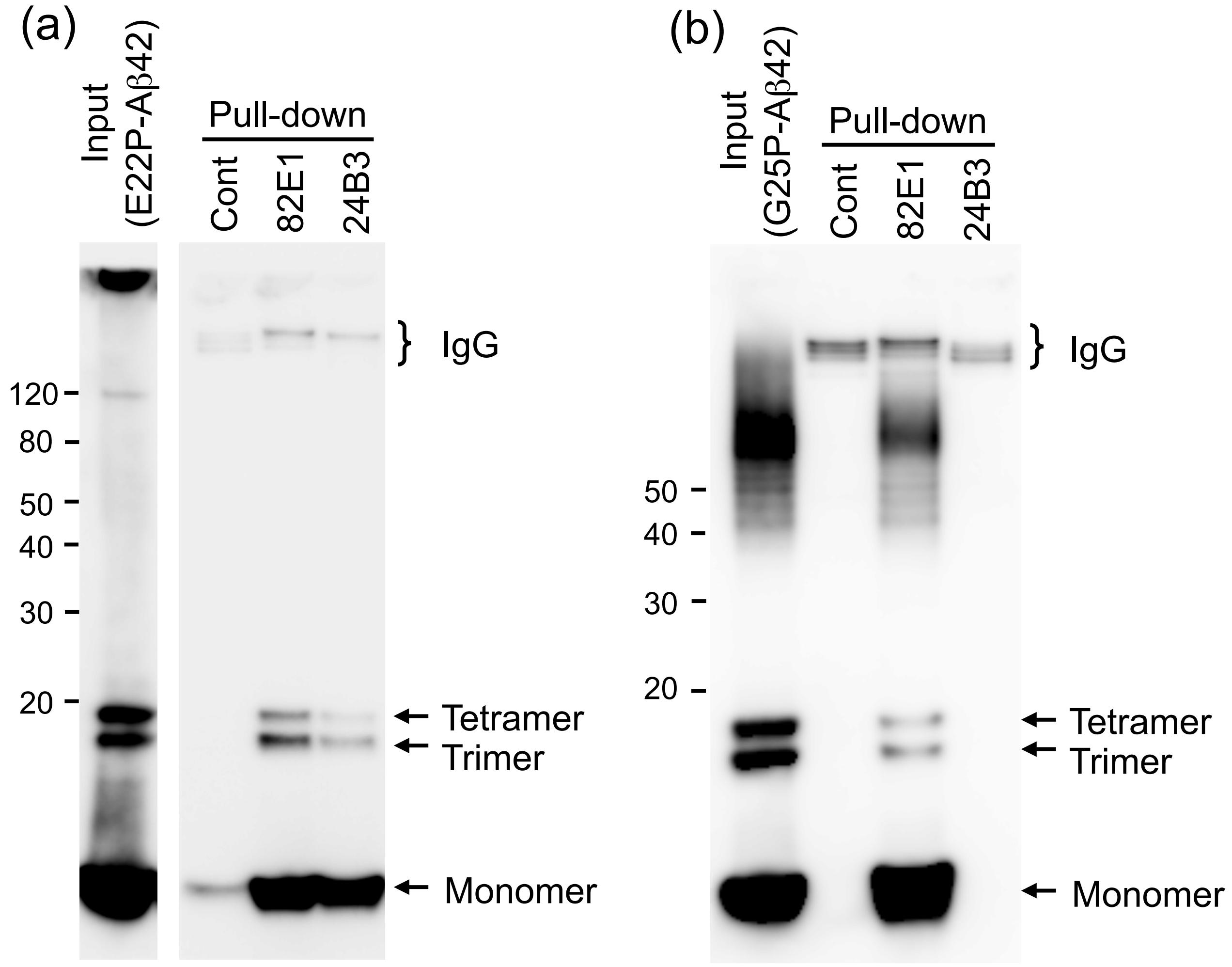

Supplementary Fig. 1. 24B3 directly binds to E22P-Aβ42, but not G25P-Aβ42. (a and b) The binding of 24B3 to E22P-Aβ42 and G25P-Aβ42 was investigated by immunoprecipitation (IP) followed by Western blotting (WB). E22P-Aβ42 solution was prepared immediately after dissolution and G25P-Aβ42 solution was prepared 24 h after dissolution for the input. No denaturing procedure (i.e. boiling or the use of detergent) was performed in the IP experiments. Mutant Aβ42 solutions underwent immunoprecipitation (IP) with control-IgG, 82E1 and 24B3. In WB, 82E1 was selected as the first antibody. In the input lane, the common bands indicated the monomer, trimer and tetramer in the mutant Aβ42 solutions; some bands were specific to each solution. In all of the lanes in WB images, high molecular weight bands, representing the IgG tetramer, were observed, indicating little protein denaturing in this IP-WB protocol. In IP with 82E1, some bands were observed in both mutant Aβ42 solutions (a and b), suggesting that 82E1 binds the toxic and non-toxic conformers. In contrast with the control-IgG, the specific bands to 24B3 were observed in E22P-Aβ42, but not in G25P-Aβ42. The bands observed in E22P-Aβ42 indicate not only monomer but also trimer and tetramer (a and b), suggesting the specific binding of 24B3 to the toxic conformer and its oligomers.

Supplementary Fig. 1

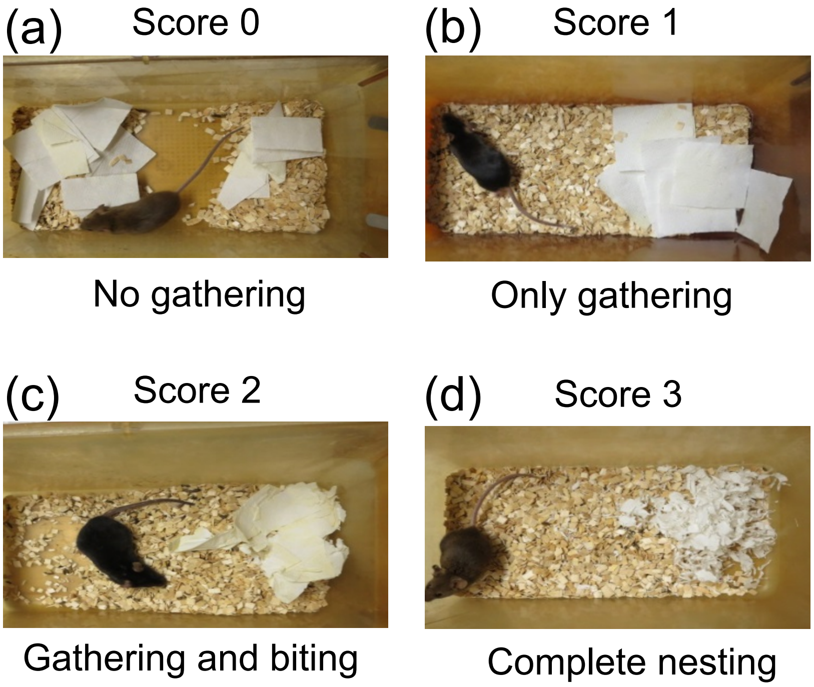

Supplementary Fig. 2. The scoring of nest construction. (a) Score 0, No gathering of the paper. (b) Score 1, Only gathering of the paper. (c) Score 2, Gathering and biting the paper. (d) Score 3, Complete nesting.

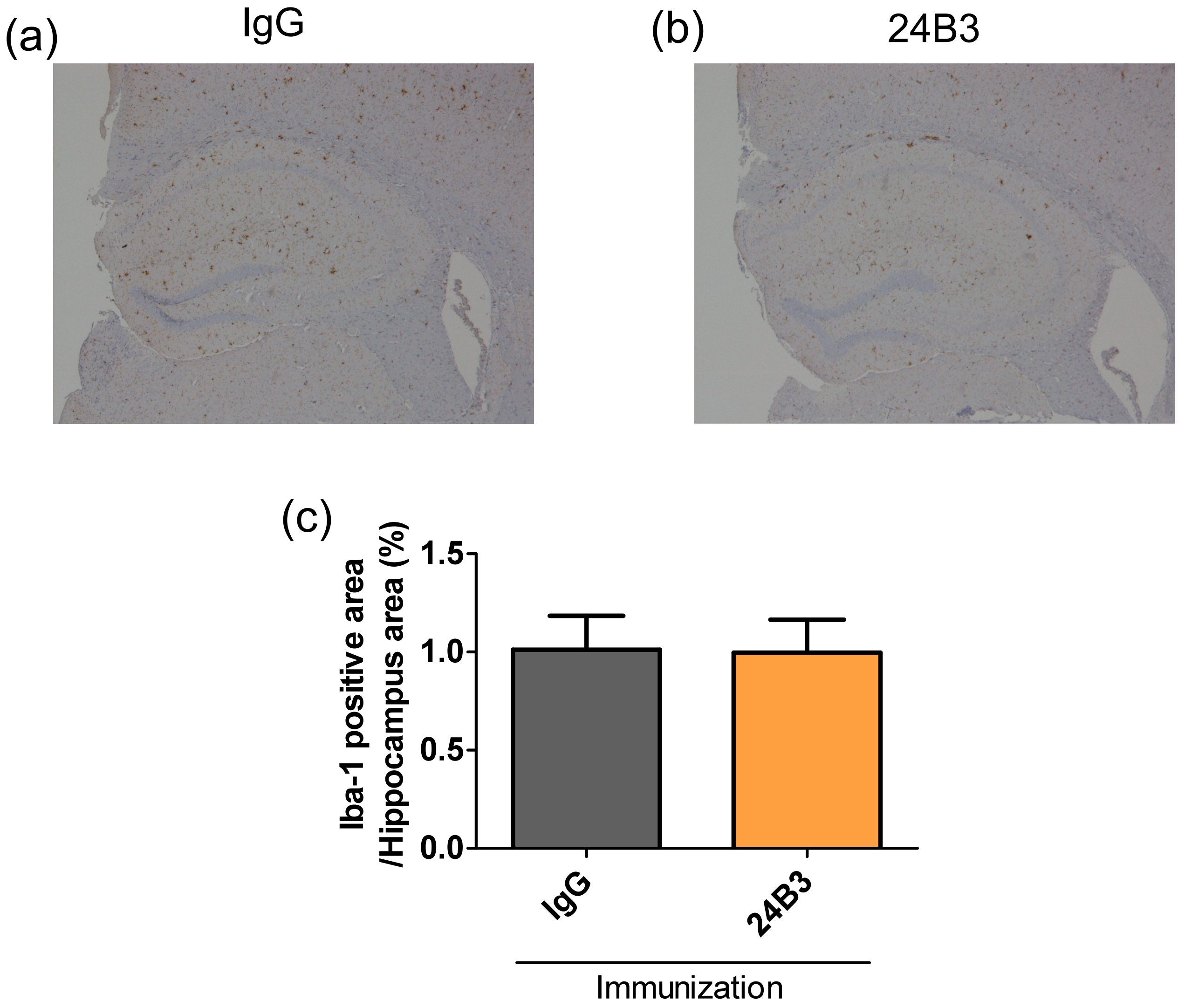

Supplementary Fig. 3. The chronic administration of 24B3 did not alter microglial activation. Representative images of immunohistochemical staining of microglia by Iba-1 on the hippocampus sections of PS2Tg2576 treated with IgG (a), and 24B3 (b). The average Iba-1 positive area is shown in the graph (c). The numbers of mice sacrificed for staining in each group were as follows: PS2Tg2576 + IgG (n = 5), and PS2Tg2576 + 24B3 (n = 6).

Supplementary Fig. 3

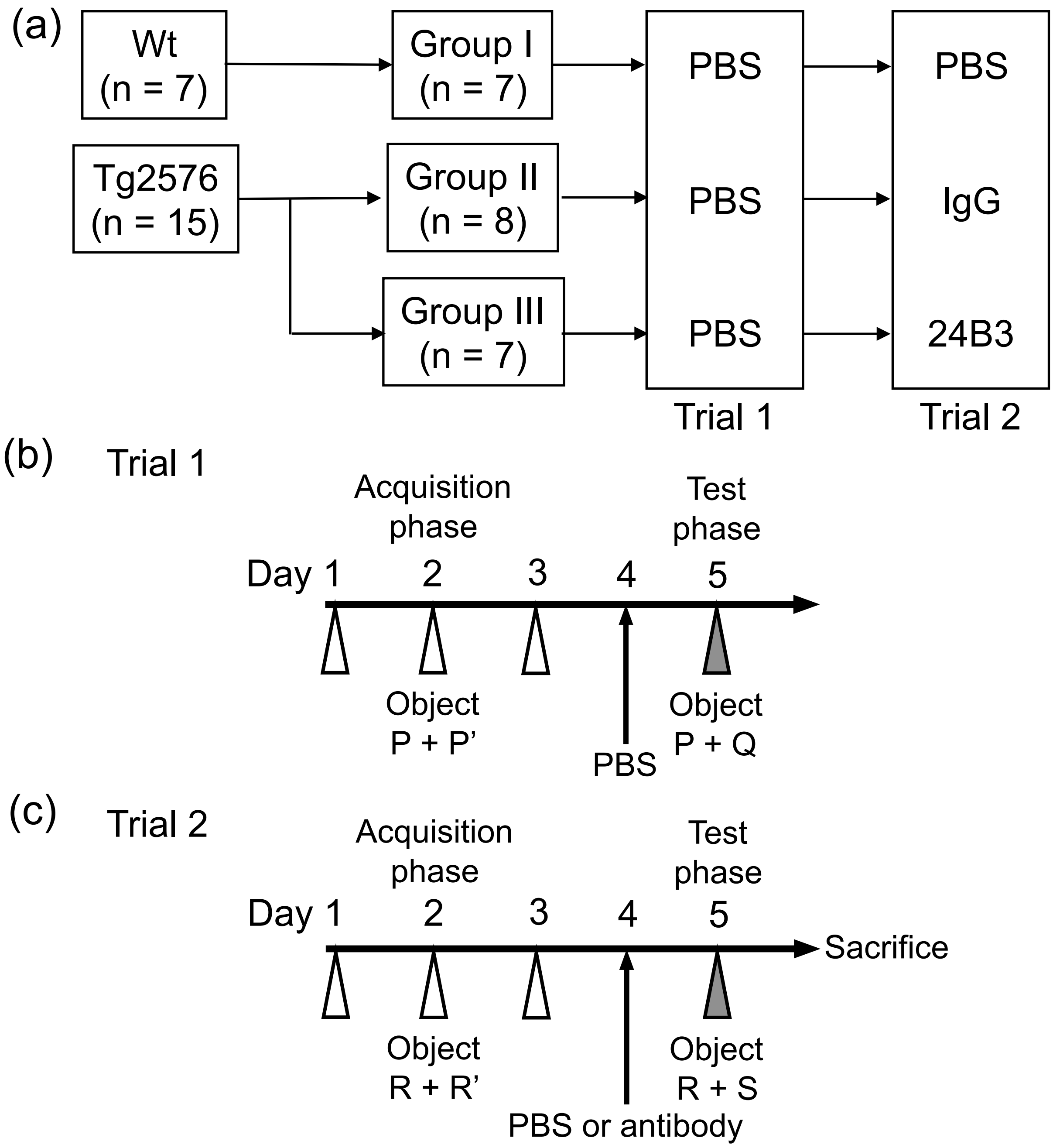

Supplementary Fig. 4. The protocols of the single administration of 24B3 and the behavioral tests. Three experimental groups (Groups I-III) were made from Wt and Tg2576. Before the test, the mice were habituated in an open experimental box for 10 min for 5 consecutive days. In this study, NOR test consisted of 2 trials. In trial 1, the mice were placed facing two similar objects (P and P') for 10 min for 3 successive days (acquisition phase), and the next day, Group I-III was intravenously injected with PBS intravenous injection. After 24 h, the mice were placed facing object P and a novel object (object Q) for 10 min (test phase). In trial 2, the mice were placed facing two similar objects (R and R') for 10 min for 3 successive days. The next day, the mice in Group I were received an intravenous injection of PBS, while the mice in Groups II and III received intravenous injections of IgG or 24B3. After 24 h, the mice were placed facing object R and a novel object (object S) for 10 min. The mice were sacrificed immediately after the experiment.

Supplementary Fig. 4
